# Emergent Magnonic Materials: Challenges and Opportunities

**DOI:** 10.3390/ma16186299

**Published:** 2023-09-20

**Authors:** Samanvaya S. Gaur, Ernesto E. Marinero

**Affiliations:** School of Materials Engineering, Purdue University, West Lafayette, IN 47907, USA

**Keywords:** magnons, CoFe alloy thin films, MgAlFeO spinel ferrites, Gilbert damping

## Abstract

Advances in information technology are hindered by energy dissipation from Joule losses associated with charge transport. In contrast, the process of information based on spin waves propagation (magnons) in magnetic materials is dissipationless. Low damping of spin wave excitations is essential to control the propagation length of magnons. Ferrimagnetic Y_3_Fe_5_O_12_ garnets (YIG) exhibit the lowest magnetic damping constants. However, to attain the lowest damping constant, epitaxial growth of YIG on single crystal substrates of Gd_3_Ga_5_O_12_ at elevated temperatures is required, which hinders their CMOS integration in electronic devices. Furthermore, their low saturation magnetization and magnetocrystalline anisotropy are challenging for nanoscale device applications. In the search for alternative material systems, polycrystalline ferromagnetic Co_25_Fe_75_ alloy films and ferrimagnetic spinel ferrites, such as MgAl_0.5_Fe_1.5_O_4_ (MAFO), have emerged as potential candidates. Their damping constants are comparable, although they are at least one order of magnitude higher than YIG’s. However, Co_25_Fe_75_ alloy thin film growth is CMOS compatible, and its magnon diffusion length is 20× longer than in MAFO. In addition, MAFO requires epitaxial growth on lattice-matched MgAl_2_O_4_ substrates. We discuss the material properties that control the Gilbert damping constant in Co_x_Fe_1−x_ alloys and MAFO and conclude that Co_x_Fe_1−x_ alloy thin films bring us closer to the realization of the exploitation of spin waves for magnonics.

## 1. Introduction

Current computing devices rely on electron transport across transmission lines and electronic devices to perform computational tasks. The flow of electrons across resistive connections results in ohmic energy losses and heat generation, thus requiring more energy to make up for efficiency losses and the need for device cooling.

Magnonics deals with the excitation, propagation, control, and detection of spin waves through a magnetic material. It is a promising field, as ohmic losses are absent. Analogous to electrons, magnons can be used as the carriers of information and their transmission without the inherent Joule losses associated with electron transport. Magnons propagate as spin waves in the material and can be launched in ferromagnets, ferrimagnets, and antiferromagnets. For comprehensive reviews on spin wave physics and devices, the reader is referred to references [1,2,3,4].

The discovery by Bertaut and Forrat [5] that yttrium iron garnet Y_3_Fe_5_O_12_ (YIG) exhibits ultralow magnetic damping parameters [5,6], α, of the order of <10^−4^ was seminal in launching the current interest in magnonics [7]. Ferromagnetic resonance (FMR) linewidth is used to measure damping parameters, and YIG exhibits the narrowest FMR linewidths and the longest spin wave propagation length [8]. With 80 atoms per unit cell, YIG is a complex crystal [8]; the attainment of the lowest damping requires epitaxial growth on single crystal substrates of gallium gadolinium garnet (GGG) using liquid phase epitaxy [9]. YIG and GGG have unit cell dimensions of 1.2376 nm and 1.2383 nm, respectively. The lattice match enables the epitaxial growth of YIG free of structural defects and strain [10]. However, deposition/annealing temperatures in the 700–850 °C range are required [11], which can promote interfacial diffusion and the formation of structural defects [12,13]. Defects impact YIG’s magnetic anisotropy, its crystal structure, and stoichiometry.

This mini review focuses on recent progress on alternative materials to YIG garnets: Co_x_Fe_1−x_ alloys and MgAl_0.5_Fe_1.5_O_4_. A brief description of spin wave dynamics using the Landau–Liftshitz–Gilbert (LLG) equation is first given. This is followed by a discussion of the material properties and mechanisms that limit the magnetic damping in these materials. The review includes an assessment of the merits of both material types for practical devices.

## 2. Magnetization Dynamics and the Gilbert Damping Constant

The dynamic response of the magnetization in a material in response to an applied magnetic field is described by the Landau–Lifshitz–Gilbert (LLG) equation [14,15], which describes the time-dependent behavior of the magnetization in response to torque forces on the magnetization.

(1) $$\frac{d\vec{M}}{dt}=-\gamma \mu_{0}\vec{M}\times {\vec{H_{e}}}_{ff}+\frac{\alpha }{M}\vec{M}\times \frac{\mathrm{d}\vec{M}}{\mathrm{d}t}$$

**Figure 1:**
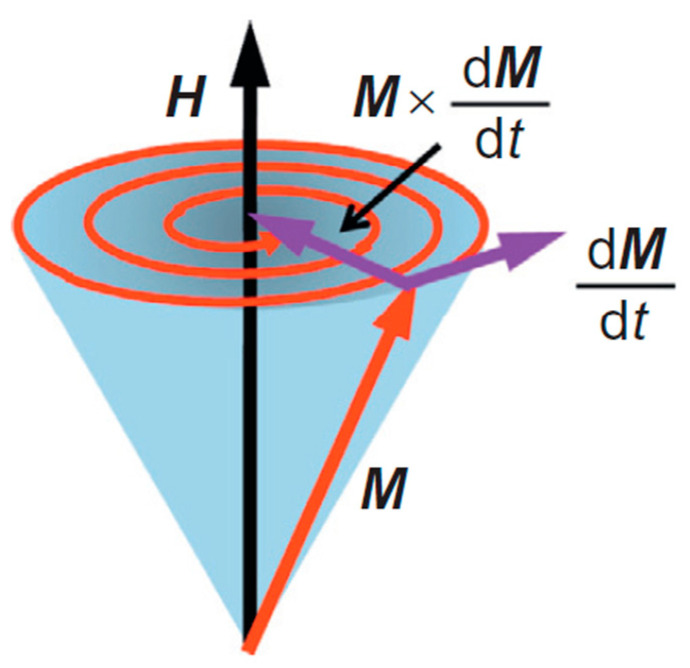
A depiction of the magnetization dynamics in response to an external magnetic field [17]. In this figure, the magnetization precesses around the applied field direction and relaxes due to the influence of magnetic damping. Reprinted with permission from Ref. [17]. Copyright 2014. Elsevier (Amsterdam, The Netherlands).

The first term represents the precession of the magnetization around the effective field *H_eff_*; this includes the applied and demagnetization fields and the anisotropy field of the material. γ is the gyromagnetic ratio, $\vec{M}$ denotes the material’s magnetization vector, and ${\vec{H_{e}}}_{ff}$ is the total effective field that acts on the magnetization. The second term represents the precessional damping, with α being a unitless constant known as the Gilbert damping constant [14]. *M*, in the denominator of the damping term, is the magnitude of the saturation magnetization as a function of time. Without damping, the magnetization of the material precesses indefinitely around the vector orientation of *H_eff_,* due to the damping term, it spirals around *H_eff_*, and it eventually aligns itself with *H_eff_*. A representation of the magnetization precessional motion is depicted in Figure 1. If the damping term is large, a long time is required for the magnetization to align with *H_eff_*. Hence, a small α value is desired for faster response times to external stimuli [16] (p. 435).

The Gilbert damping constant is a material’s limiting factor for magnon propagation. The equation also indicates that a larger saturation magnetization $\vec{M}$ (the projection of *M*, i.e., cosθ times magnitude of *M*) can counterbalance, within limits, the effect of the damping constant. In fact, experimentally, it is found that despite the fact that MAFO and Co_25_Fe_75_ have comparable Gilbert damping constants, Co_25_Fe_75_ alloy thin films exhibit larger magnon propagation lengths (~20 µm) [18] than MgAl_0.5_Fe_1.5_O_4_ (~0.8 µm) [19]. This is largely attributed to the larger saturation magnetization moment of Co_25_Fe_75_ of 2.4 T [20], in contrast to 0.1256 T [21] for MgAl_0.5_Fe_1.5_O_4_.

**Figure 2:**
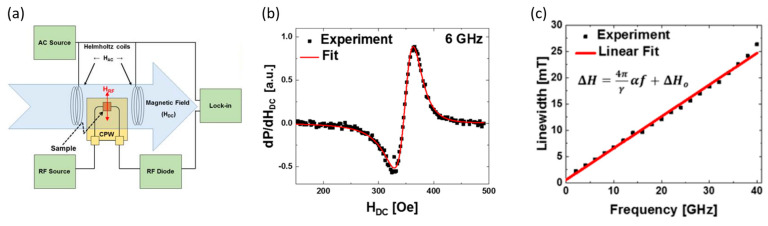
(**a**) Schematic of an experimental setup employed for measurements of the Gilbert damping parameter; the key components of an FMR apparatus are identified. (**b**) Shows a representative spectrum of the derivative of transmitted RF power (dP/dH_DC_) vs. swept magnetic field (H_DC_). (**c**) Linewidth vs. RF frequency from 2 to 40 GHz [22]. Note the excellent fit with the experimental data. Reprinted with permission from Ref. [22]. Copyright 2023. Quantum Design Inc. (San Diego, CA, USA).

The Gilbert damping parameter is derived from Ferromagnetic resonance (FMR) measurements [22]. Figure 2a provides a schematic representation of the FMR setup.

In the FMR measurement instrumentation, the coplanar waveguide (CPW) provides radio frequency (RF) microwave signals to the sample over a broad range of frequencies (2–40 GHz). The microwaves generate magnetic fields at RF frequencies (H_RF_), which can resonantly excite the magnetic samples at specific magnetic fields (H_DC_) and frequencies, which depend on the material properties. The magnetic thin films are placed film side down on the CPW, as the RF magnetic fields do not extend far from the interface between the thin film and the CPW. Therefore, this maximizes coupling and signal output. Furthermore, the sample should not short out the CPW. To this effect, the sample is often coated with an insulating layer, or the CPWs can also be insulated with a single layer of transparent tape. As shown in Figure 2a, H_RF_ should be perpendicular to H_DC_ in order to provide the most efficient excitation mechanism. To eliminate the frequency-dependent background response that may mask the relatively weak FMR response of the sample, measurements are typically performed at a fixed frequency while sweeping H_DC_. The sample magnetization undergoes a resonant response as H_DC_ is swept through the resonance condition, absorbing CPW energy. By sweeping through the resonance field, RF energy is reduced, which is converted to DC voltage by a broadband RF diode.

Lock-in detection is used to improve the signal-to-noise ratio (SNR), which involves modulating the signal at a known frequency. An additional set of Helmholtz coils powered by an AC source produces a small (~1 Oe) modulation (H_AC_) to the much larger H_DC_. In this scheme, the derivative of transmitted power (dP/dH_DC_) is actually measured, as schematically illustrated in Figure 2b. We refer the reader to reference [22] to learn how linewidth ΔH is obtained for a particular frequency.

When the ΔH data are plotted vs. the RF frequencies (GHz), a plot of ΔH vs. f, as shown in Figure 2c, is obtained. The equation in the inset of Figure 2c is employed to extract the Gilbert damping parameter α. The higher the slope of ΔH vs. f, the higher the value of the total damping constant, α.

## 3. Ferromagnetic Co_1−x_Fe_x_ Alloy Thin Films

**Figure 3:**
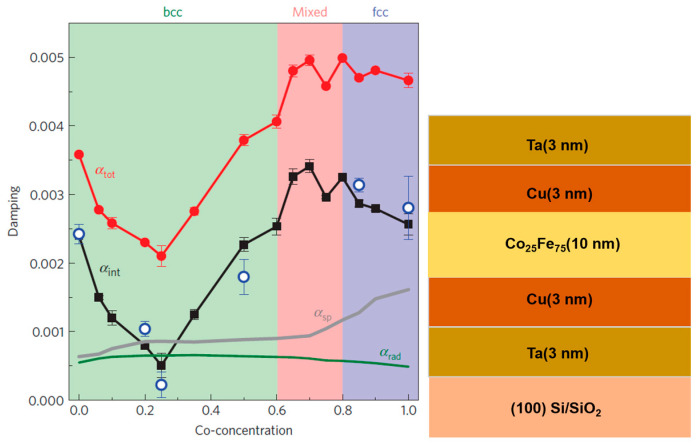
Plot of Gilbert damping parameter vs. Co composition in Co_1−x_Fe_x_ thin films [20]. Note that at 25% Co composition, there is a sharp decrease in the damping parameter (both intrinsic and total). On the right side of the Figure, the thin film stack employed is illustrated. Reprinted with permission from Ref. [20]. Copyright 2016. Springer Nature (Berlin, Germany).

Metallic ferromagnetic films for magnonics have neglected, as it is assumed that they are unlikely to exhibit low magnetic damping due to magnon–electron scattering by the conduction electrons. Schoen et al. [20] reported on the composition dependence of the Gilbert damping constant of polycrystalline 10 nm thick Co_1−x_Fe_x_ thin films grown at room temperature by sputter deposition onto Cu(3 nm)/Ta(3 nm) seed layers. The thin films were also capped with Cu(3 nm)/Ta(3 nm) bilayers. Their results are reproduced in Figure 3, indicating that the alloy with 25 at. % Co exhibits the lowest damping parameter.

**Figure 4:**
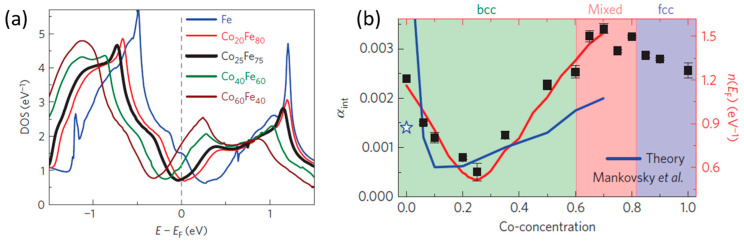
(**a**) Correlation of the minimum damping at 25% Co composition and the electronic density of states (DOS). Bulk electronic structure obtained for various Co_1−x_Fe_x_ alloys are shown in different colors. Note that the dashed vertical line at 0 is *E_F_* to ease the comparison. (**b**) Plot of intrinsic damping of Co_1−x_Fe_x_ (different Co compositions) compared with theoretical calculations by Mankovsky et al. [23]. Note the deep minimum in the density of states at Fermi energy n(E_F_) [20]. Reprinted with permission from Ref. [20]. Copyright 2016. Springer Nature.

The measured values of the total damping constant for 10 nm thin films are α_total_ = (2.1 ± 0.1) × 10^−3^ at a Co-concentration of 25%. As discussed by the authors, extrinsic contributions increment the value of the intrinsic damping constant, α_int_. Two significant effects are radiative damping and spin pumping. Radiative damping arises from inductive coupling of the precessing magnetization and the coplanar waveguide employed in the FMR measurement. The damping contribution due to spin pumping involves spin polarized electron injection from CoFe into the Cu/Ta seed and capping layers. Figure 3 provides intrinsic damping constant values after correction for extrinsic contributions. An ultralow value of α_int_ = (5 ± 1.8) × 10^−4^ at a Co-concentration of 25% is reported. The strong composition dependence of the Gilbert damping constant in Co_1−x_Fe_x_ alloys is ascribed by Schoen et al. to changes in the electron density of states (DOS) at the Fermi level as a function of composition. Their electronic structure calculations, together with the values of α_int_, are shown in Figure 4 and clearly indicate that the damping is smallest at the DOS minimum for Co = 25%. For full details and explanation of this Figure, the reader is referred to reference [20].

Experiments by Fackle et al. [18] on sputter deposited thin films of Co_25_Fe_75_ alloy confirmed the ultralow values of its intrinsic damping parameter (α_int_ = (3.18 ± 0.48) × 10^−4^). They employed an out-of-plane (hard axis) FMR measurement geometry to suppress two-magnon scattering contributions to the damping parameter [24]. The intrinsic damping constant is impacted by radiative, spin pumping, and spin-flip processes, resulting in values that exceed the intrinsic value by 4.2×.

**Figure 5:**
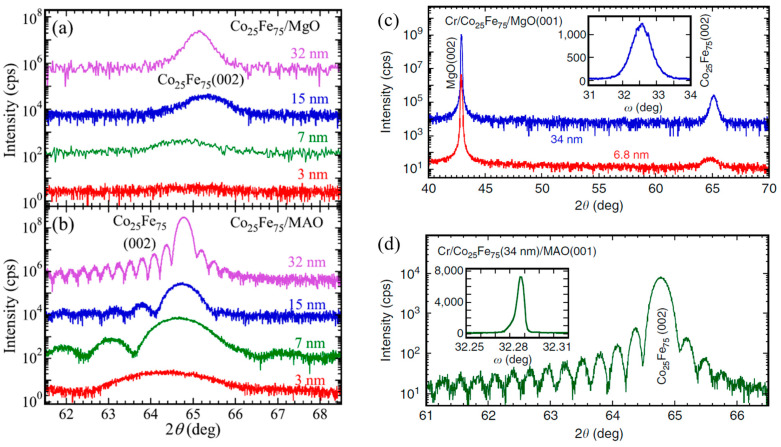
XRD spectra of Co_25_Fe_75_ thin films with varying thicknesses grown on (**a**,**c**) MgO and (**b**,**d**) MgAl_2_O_4_ (MAO) [25,27]. Note the Laue oscillations for films grown on MAO, which are indicative of high-quality crystalline growth. Insets in (**c**,**d**) are XRD rocking curve measurements; the lower full width at half maxima (FWHM) is indicative of less mosaic spread and a better degree of epitaxy in the case of films grown on MAO. Figure 5a,b reprinted with permission from Ref. [25]. Copyright 2018. AIP Publishing (College Park, MD, USA).

The intrinsic damping constant in Co_25_Fe_75_ alloys is also controlled by the thin film microstructure; the role of epitaxial growth has been investigated by Cheng et al. [25], and the influence of the seed/buffer layers has been investigated by Edwards et al. [26]. A comparison of thin film growth quality on MgO and MAO substrates is presented in Figure 5. The lattice parameters of the Co_25_Fe_75_ film and the MAO substrates have a lattice mismatch of 0.4% compared to 3.9% with MgO [25,27]. Thus, epitaxial growth of Co_25_Fe_75_ films on MAO substrates is achieved as opposed to strained epitaxial growth of Co_25_Fe_75_ growth on MgO substrates. Figure 5a shows XRD spectra for films of Co_25_Fe_75_ of various thicknesses grown directly on (001) MgO substrates with 2.8 nm Cr capping layers. Similarly, Figure 5b presents XRD spectra for films of Co_25_Fe_75_ of different thicknesses grown on (001) MAO substrate with the same capping layers. The films grown on MAO exhibit superior crystalline quality compared to those grown on MgO. This is confirmed by the observation of Laue oscillations on Co_25_Fe_75_ films grown on MAO with a thickness ≥ 7 nm [25,27].

Similar structural results were observed by Lee et al. [27] Figure 5c presents XRD measurements for Co_25_Fe_75_ films (6.8 nm and 34 nm) grown directly on MgO with 2.8 nm Cr capping layers, whereas corresponding measurements for 34 nm Co_25_Fe_75_ thin films grown on MAO are shown in Figure 5d [27]. Laue oscillations are also observed for growth on MAO substrate, which are indicative of the excellent crystal quality of the thin film. The rocking curve measurements (see insets in (c,d)) exhibited FWHM values of 0.68^0^ for films grown on MgO compared to 0.0057^0^ for films grown on MAO.

**Figure 6:**
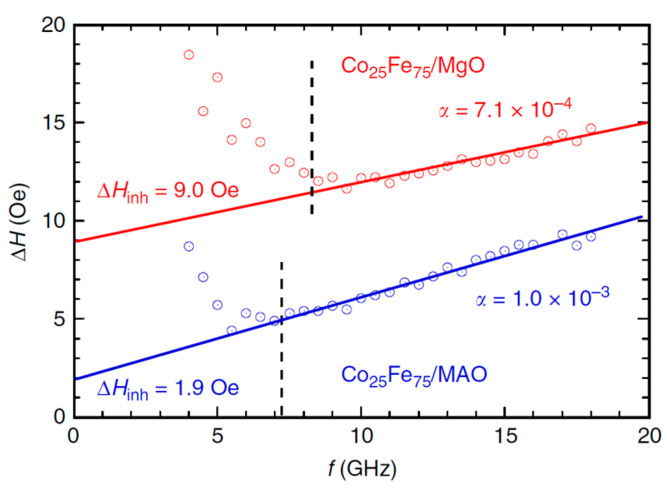
Ferromagnetic resonance linewidths (Oe) vs. frequency (GHz) of Cr (2.8)/Co_25_Fe_75_(6.8)/MgO(001) in red and Cr(2.8)/Co_25_Fe_75_(6.8)/MgAl_2_O_4_(001) in blue. Solid lines represent linear fit for data points represented by circles. Dashed lines represent frequencies below which inhomogeneous broadening takes place due to incomplete saturation of the films [27]. Numbers in brackets are thicknesses in nm. Note that lower slope ΔH/f indicates lower damping.

Figure 6 provides FMR linewidth measurements vs. frequency for samples grown on MgO and MAO [27]. The total damping for Cr(2.8 nm)/Co_25_Fe_75_(6.8 nm)/MgO was calculated as 0.71 × 10^−3^, and that for Cr(2.8 nm)/Co_25_Fe_75_(6.8 nm)/MAO was 1 × 10^−3^. These results are somewhat surprising, as Co_25_Fe_75_ films grown on MAO exhibit superior crystalline growth quality than those grown on MgO. One would expect a higher degree of crystalline disorder (grain boundaries) to negatively contribute to the damping parameter, yet the opposite is observed here. Other thin film properties not reported in this study, such as film roughness, could contribute to the differences reported. Further thin film structural characterization is required to explain these interesting results.

## 4. Role of Interfaces on the Damping Constant in Co_25_Fe_75_ Thin Films

**Figure 7:**
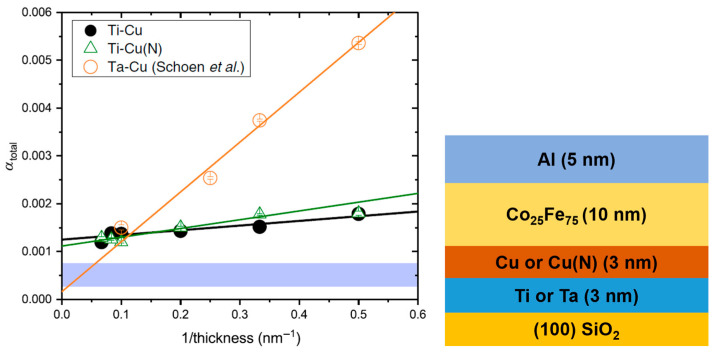
Total damping measurements (using out-of-plane FMR geometry) in Co_25_Fe_75_ films of different thicknesses deposited on various combinations of 3 nm seed/3 nm buffer layers [26]. The blue region indicates the intrinsic damping measurements from reference [20]. On the right-hand side of the Figure, the thin film stack used is depicted. Ti-Cu(N) means that the stack consists of a 3 nm Ti seed layer and a 3 nm Cu (Nitrogen doped) buffer layer. All samples were capped with 5 nm of Al. Reprinted with permission from Ref. [26]. Copyright 2019. American Physical Society (College Park, MD, USA).

Spin pumping impacts damping, and it depends on the nature of the non-magnetic layers in contact with the Co_25_Fe_75_ thin films. Edwards et al. [26] studied the effect of 3 nm seed (Ti, Ta)/3 nm buffer (Cu, Cu(N)) bilayers on damping of Co_25_Fe_75_ thin films of different thicknesses. Figure 7 presents the measured total damping measurements. The film stacks employed in their work are also given. It was found that when Co_25_Fe_75_ thin films were grown on Ti seed layers, the spin pumping contribution was minimized, as Ti is not a good absorber of spin currents.

**Figure 8:**
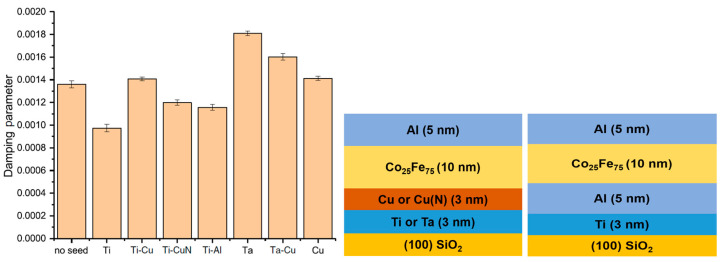
The total damping constant of 10 nm thick Co_25_Fe_75_ thin film grown on different seed/buffer layer combinations. All seed layers are 3 nm thick, except for Al (5 nm) [26]. Notation: Ti, Cu (Ti, Cu seed layer, no buffer layer); Ti-Cu, Ti-CuN, Ti-Al, Ta-Cu (Ti or Ta seed layer, Cu, CuN, Al buffer layer). Reprinted with permission from Ref. [26]. Copyright 2019. American Physical Society.

Figure 8 shows the effect of the nature of the buffer layer on the damping constant of 10 nm thick Co_25_Fe_75_ thin films. The 3 nm thick Cu, Cu(N) buffer layers were grown on 3 nm Ti and Ta seed layers, as well as on 5 nm Al buffer layers on 3 nm Ti seed layers. All samples were capped with 5 nm Al layers. The various combinations are schematically illustrated in the figure.

The structure with a Ti seed layer without a buffer layer exhibited the lowest total damping constant, and Ti-X bilayers exhibited lower damping than Ta-X. This is indicative that spin pumping contributions are effectively suppressed by utilizing Ti seed layers [26].

## 5. Role of Interlayer Thickness on the Damping Constant in Co_25_Fe_75_ Thin Films

**Figure 9:**
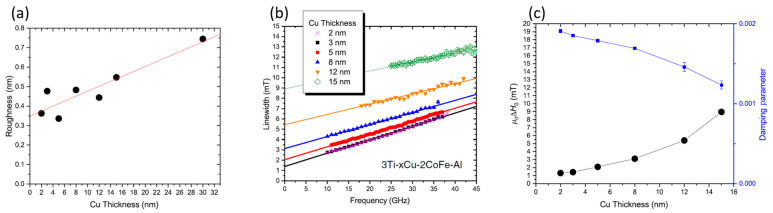
(**a**) Variation of thin film roughness (AFM measurements) vs. Cu buffer layer thickness. (**b**) FMR linewidth vs. Cu buffer thickness from 2 to 15 nm. Note that a smaller slope is indicative of lower total damping. (**c**) Damping parameter variation with Cu thickness [26]. Reprinted with permission from Ref. [26]. Copyright 2019. American Physical Society.

The thickness of the seed/buffer layers (interlayers) influences the thin film roughness. The effect of roughness on the magnetic damping parameter was studied by Edwards et al. [26] in Ti(3 nm)/Cu(x)/Co_25_Fe_75_(2 nm)/Al(5 nm) stacks by varying the Cu buffer layer thickness. Film roughness introduces magnetic inhomogeneities that negatively impact the damping constant [28,29]. Figure 9a shows that as the thickness of the Cu layer increases, the RMS roughness of the film stack significantly increases. Figure 9b provides the linewidth vs. frequency measurements for these films, whose slopes are used to calculate the total damping constant for the stacks. In Figure 9c, the dependence of the total damping parameter on Cu buffer layer thickness is provided, and it exhibits a clear trend: as the Cu buffer layer thickness increases, the thin film stack RMS roughness increases, which, in turn, reduces the total damping. This result is also surprising, as interlayer roughness is expected to negatively impact magnon propagation. Further structural studies are needed to understand these somewhat puzzling results.

## 6. MgAl_2−x_Fe_x_O_4_ Spinel Ferrites

MgAl_2−x_Fe_x_O_4_ (MAFO), where x < 2, is a ferrimagnetic material in which Fe occurs in Fe^2+^ and Fe^3+^ states and is responsible for the overall magnetization of the material. There are 56 atoms per unit cell in MAFO, of which O^2−^ anion coordinated tetrahedral sites are occupied by Mg^2+^ and half of the Fe^3+^ cations, whereas O^2−^ anion coordinated octahedral sites are occupied by Al^3+^, Fe^2+^, and half of the Fe^3+^ cations [11]. Thin films of MAFO can be epitaxially grown by pulsed laser deposition (PLD) on single crystal spinel MgAl_2_O_4_ (MAO) substrates as their lattice mismatch is small (2%) [11]. The MgAl_0.5_Fe_1.5_O_4_ spinel ferrite exhibits an ultralow damping parameter of the order of 1.5 × 10^−3^, which is also associated with a minimum density of states that arises due to suppression of intraband electronic transitions. The low density of states at the Fermi level implies fewer conduction electrons, i.e., low damping.

## 7. The Role of Lattice Matching on Damping in MAFO

The close lattice match between MAFO and MAO is critical to attain low magnetic damping as it eliminates the formation of structural defects. Structural defects, such as dislocations, and the presence of antiphase boundaries increase magnetic damping [30,31,32]. Defects also lead to inhomogeneous contributions to damping by creating localized nonuniform magnetization [33]. Thin MAFO films with uniform magnetic properties grown on lattice-matched substrates are required to minimize damping contributions from structural defects.

In the case of MAFO, the importance of coherent strain over partial strain relaxation has been shown to result in low damping [11]. Recent studies of the growth of MgAl_0.5_Fe_1.5_O_4_ films on single crystal MgAl_2_O_4_ substrates to obtain coherent strain report damping parameters of α ~ 0.001–0.002 [21,34,35,36].

## 8. The Role of Film Thickness on Damping in MAFO

**Figure 10:**
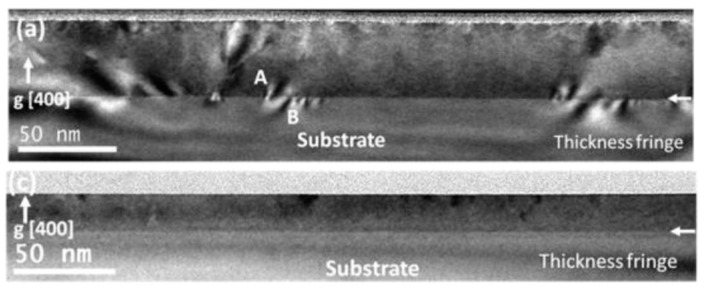
Bright-field TEM images of MgAl_0.5_Fe_1.5_O_4_; (**a**) 40 nm and (**c**) 18 nm (the horizontal white arrow shows the film–substrate interface) [21]. Note the higher number of defects present in the thicker film (regions A and B in (**a**)), which are ascribed to partial stress relaxation of the film. The thinner film remains coherently strained. Reprinted with permission from Ref. [21]. Copyright 2018. American Chemical Society (Washington, DC, USA).

Emori et al. [21] used pulsed laser deposition (PLD) to grow MgAl_0.5_Fe_1.5_O_4_ thin films on MgAl_2_O_4_ substrate to study the thickness dependence of the damping parameter. The bright-field TEM images of Figure 10 show that the thicker 40 nm films exhibit many defects arising primarily from strain relaxation. Such defects are absent in the 18 nm films.

**Figure 11:**
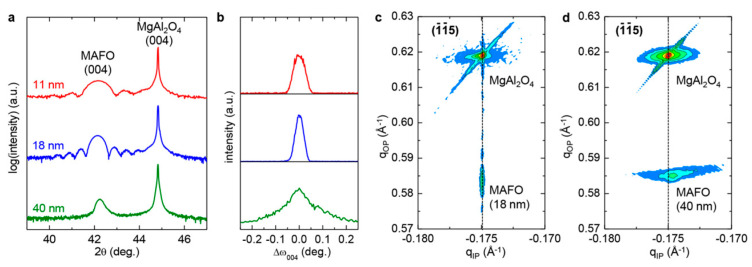
Structural characterization of MgAl_0.5_Fe_1.5_O_4_ film with different thicknesses grown on MgAl_2_O_4_ substrate: (**a**) Combined 2θ XRD plots for films with varying thicknesses. (**b**) Rocking curves for the same films. (**c**,**d**) Reciprocal space maps for films of thicknesses of 18 nm and 40 nm, respectively [21]. Reprinted with permission from Ref. [21]. Copyright 2018. American Chemical Society.

XRD, rocking curve measurements, and reciprocal space maps validate the role of film thickness on crystalline quality. The XRD results reproduced in Figure 11a indicate that MAFO films with thicknesses < 20 nm exhibit a higher degree of crystallinity, as evidenced by the presence of Laue oscillations that arise from the smooth texture of the film [21,37]. The rocking curve measurements from Figure 11b corroborate these findings. The full width at half maximum (FWHM) of the (004) peak for MAFO films < 20 nm thick is ~0.045–0.06° as compared to ~0.2° for 40 nm thick films [21]. Also, no Laue oscillations are observable in the 40 nm thick films, indicative of a poorer degree of crystallinity.

Additional structural differences between MAFO films with varying thicknesses are provided by reciprocal space maps given in Figure 11c,d [21]. For the 18 nm thick MAFO films, there is virtually no mosaic spread. On the other hand, the 40 nm thick films exhibit a large mosaic spread; this is consistent with the TEM images (i.e., the 18 nm films are coherently strained to the substrate, whereas the 40 nm films are partially relaxed due to the presence of defects near the substrate interface).

**Figure 12:**
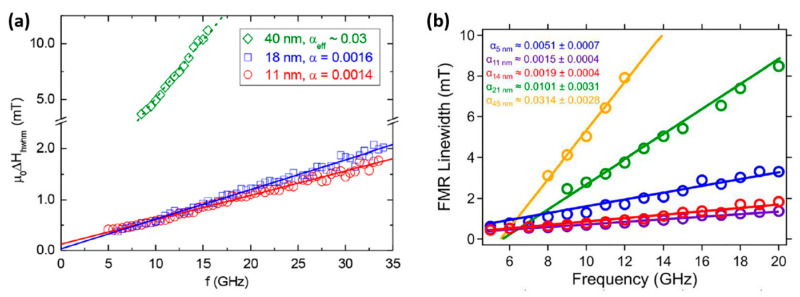
FMR linewidth vs. frequency. (**a**) MgAl_0.5_Fe_1.5_O_4_ films with thicknesses of 11 nm, 18 nm, and 40 nm, respectively [21]; (**b**) MgAl_0.5_Fe_1.5_O_4_ films with thicknesses of 5 nm, 11 nm, 14 nm, 21 nm, and 45 nm, respectively [38]. Figure 12a reprinted with permission from Ref. [21]. Copyright 2018. American Chemical Society. Figure 12b reprinted with permission from Ref. [38]. Copyright 2019. AIP Publishing.

FMR measurements were utilized to correlate the structural properties of MAFO films with different thicknesses to magnetic damping parameters. In Figure 12a, α for the 11 nm and 40 nm thick films are 0.0014 and ~0.03, respectively. Similar results are shown in Figure 12b; note, however, that damping significantly increases in the 5 nm thick films. This increase in damping is attributed to the presence of a ~1 nm thick magnetic dead layer at the interface of the MAFO film and substrate. Such a layer is the region of chemical disorder that is iron deficient and that negatively affects the magnetic properties of 5 nm MAFO films. These results indicate that damping is directly correlated to the microstructural properties of MAFO films, which are influenced by the thickness of the magnetic film.

## 9. The Role of Fe Content in MAFO on Magnetic Damping Constant

**Figure 13:**
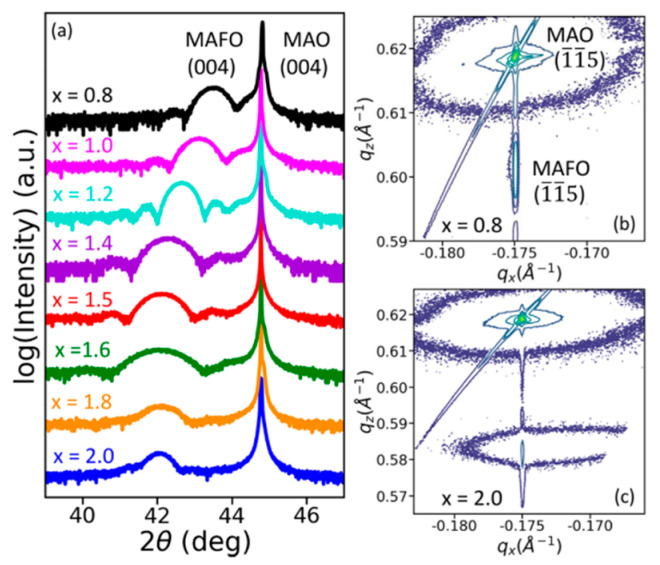
Structural characterization of 11 nm thick MgAl_2−x_Fe_x_O_4_ films with varying Fe content (x = 0.8 to x = 2). (**a**) Combined XRD plots of different films with different Fe content, (**b**,**c**) Reciprocal space maps for MgAl_1.2_Fe_0.8_O_4_ and MgFe_2_O_4_ respectively [36]. Reprinted with permission from Ref. [36]. Copyright 2020. AIP Publishing.

The Fe content in MgAl_2−x_Fe_x_O_4_ influences the magnetic damping properties. In Figure 13a, Laue oscillations around (004) are clearly seen in the XRD spectra for films with x < 1.6, indicating good crystallinity [36]. However, no Laue oscillations are present in films with higher Fe content (i.e., x > 1.5), which is indicative of poorer crystalline quality.

**Figure 14:**
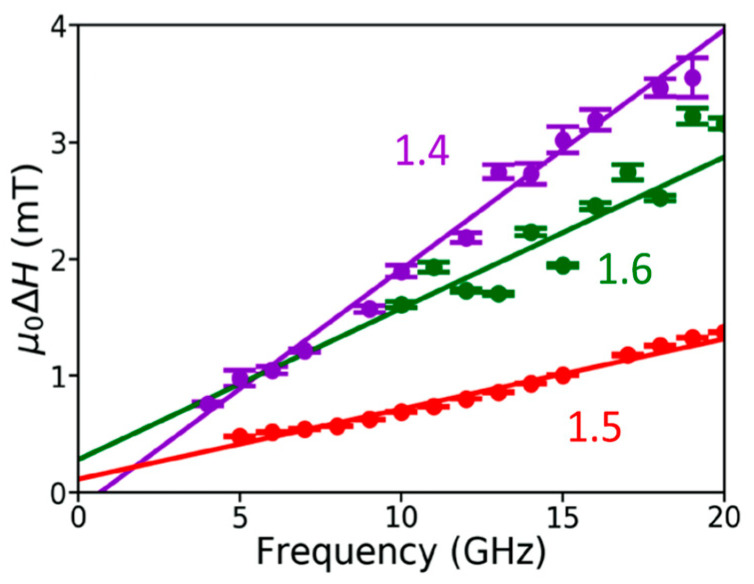
FMR linewidth, dH, vs. frequency for MgAl_2−x_Fe_x_O_4_ films with varying iron content: MgAl_0.6_Fe_1.4_O_4_, MgAl_0.5_Fe_1.5_O_4_, and MgAl_0.4_Fe_1.6_O_4_ [36]. Reprinted with permission from Ref. [36]. Copyright 2020. AIP Publishing.

The reciprocal space maps of Figure 13b,c indicate excellent lattice matching and low mosaic spread for MgAl_1.2_Fe_0.8_O_4_ as opposed to poor lattice matching and large mosaic spread for MgFe_2_O_4_. Films with x < 1.4 have Curie temperatures (T_C_) below room temperature and, hence, were neglected in this study [36]. When the Fe content x > 1.6, the coercivity increases, possibly due to incoherent film growth. Films with 1.4 ≤ x ≤ 1.6 show soft magnetism or ferrimagnetism [36]. As seen in Figure 14, MgAl_0.5_Fe_1.5_O_4_ shows the narrowest FMR linewidth and the lowest damping constant (1.8 ± 0.01) × 10^−3^ [36]. For higher iron content (x > 1.6), the film quality degrades and the coercivity increments. This is attributed to magnetic frustration and defect pinning. Thus, the ideal iron content range was concluded to be 1.4 ≤ x ≤ 1.6 [36].

## 10. Discussion

The material parameters that affect the Gilbert damping parameter in ferromagnetic Co_25_Fe_75_ thin films and ferrimagnetic MgAl_0.5_Fe_1.5_O_4_ have been discussed in this review. Neither system attains the ultralow magnetic damping of YIG garnets. However, for VLSI devices, magnon propagation lengths in the sub to tens of micron regime are of interest.

Table 1 compares key parameters for magnonics (Gilbert damping constant and magnon propagation distance) as well as fabrication challenges for YIG, Co_25_Fe_75_, and MgAl_0.5_Fe_1.5_O_4_. YIG exhibits ultra-low magnetic damping constants of ~10^−5^, resulting in magnon propagation lengths of the order of centimeters. While highly desirable for magnonics, YIG growth requires liquid phase epitaxy and high temperatures (~1000 °C) and single crystal GGG substrates, which makes YIG garnets presently unsuitable for CMOS integration. Thus, the major challenges that YIG garnets need to overcome are to identify alternate growth techniques and suitable, low-cost substrates for CMOS integration.

In the case of Co_25_Fe_75_, α is of the order of 10^−3^ and the magnon propagation length is ~20 µm. The advantages of this material system are film growth by sputter deposition, a widely used method in industry, growth conducted at an ambient temperature on Si wafers, and no post-processing treatments required. These alloys are widely employed in magnetic recording, and the engineering of their magnetic and structural properties is well established. Attainment of the lowest magnetic damping parameters requires careful selection of ancillary materials (seed and buffer layers) to eliminate extrinsic factors that negatively contribute to damping.

The magnetocrystalline anisotropy in magnetic materials derives from spin-orbit coupling and results in preferential orientation of the magnetization along specific crystallographic directions in the material. Thus, one needs to address the impact of the magnetocrystalline anisotropy in the Co_25_Fe_75_ on the spin wave propagation. Kroner et al. fabricated a magnonic device based on Co_25_Fe_75_ and measured a saturation magnetization (Ms) of ~2.4 T. FMR measurements were employed to estimate the “effective magnetization,” which is the difference between the in-plane saturation magnetization and the perpendicular anisotropy field. The value derived for (M_s_) was ~2.4 T, and FMR measurements were employed to estimate the difference between the in-plane saturation magnetization and the perpendicular anisotropy field. The derived value for (M_eff_) is ~1.91 T, and, for the perpendicular anisotropy field, $H_{u}^{⊥}$ ~ 0.49 T based on the relation $Meff=Ms-H_{u}^{⊥}$ [42]. In addition, FMR measurements revealed that the sample was almost magnetically isotropic with uniaxial in-plane (IP) anisotropy, $H_{u}^{IP}$~17 Oe [1.7 mT], which is oriented along the (110) direction. A fourfold symmetry is expected in bcc Co_25_Fe_75_; the weak in-plane anisotropy is indicative of the polycrystalline growth of the sample. The authors conducted spin wave propagation experiments via Time-Resolved Magneto Optic Kerr Effect (TRMOKE), both including the contribution of small uniaxial IP anisotropy and neglecting it. Interestingly, it was found that the spin wave propagation lengths 5–8 μm were almost identical for both cases. In a different study by Schoen et al., similar perpendicular magnetic anisotropy, $H_{u}^{⊥}$, was observed for Co_25_Fe_75_ as a result of the small thickness of the Co_25_Fe_75_ sample and the contribution of interfacial anisotropy at the interfaces of Co_25_Fe_75_ and the cap and seed layers [20]. This anisotropy can be easily tuned by choosing optimal seed and cap layers with the material of interest.

In the case of MgAl_0.5_Fe_1.5_O_4_ thin films, the damping constant is of the order of 10^−3^, which is comparable to that of Co_25_Fe_75_. However, the magnon diffusion length is anisotropic (crystalline orientation dependent) and ranges from 0.6 to 0.9 µm. This is considerably less than in Co_25_Fe_75_ (~20 µm) on account of MAFO lower saturation magnetization. MAFO is grown by pulsed laser deposition on single crystal MAO substrates. The substrates need to be heated to ~450 °C. Key challenges with this system are the scalability of PLD growth for large sample and volume fabrication and the cost of the MAO single crystal substrates.

The exchange interactions in magnetic materials determine the spin alignment of the electrons responsible for their magnetism. The strength of the exchange interactions is defined by intrinsic material properties: the saturation magnetization (Ms) and the exchange stiffness constant (A). A is dependent on the electronic structure of the constituent atoms and their nearest neighbor spacing. The magnetostatic exchange length is calculated using the expression $Lex=\sqrt{\frac{A}{2\pi {Ms}^{2}}}$ [43]. Exchange length values estimated using this expression are: YIG (~17.6 nm) [41], Co_25_Fe_75_ (~3.4 nm) [42], and MgAl_0.5_Fe_1.5_O_4_ (~20.5 nm) [19]. As indicated in reference [44], spin wave characteristics are defined by two types of interactions: strong short-distance exchange interactions and weak long-range dipole–dipole interactions. The corresponding spin wavelengths are <1 µm for exchange interactions, whereas for the dipolar interactions, the spin wavelengths are >1 µm [44]. The spin propagation length in Co_25_Fe_75_ is reported to be >20 µm [18]; the authors attribute such long propagation lengths to its low damping properties in combination with its high saturation magnetization, resulting in long-range propagation of dipolar spin waves. These spin wave characteristics are appropriate for the material to be considered as a promising candidate for nanoscale magnonic devices.

We suggest that ferromagnetic Co_25_Fe_75_ alloys are the most promising alternative materials to YIG for magnonic applications given the relatively easy method of fabrication and the large magnon propagation lengths. Their large, tunable saturation magnetization is an effective tool for reducing the contribution of the damping term (LLG Equation (1)) to the magnetization precession, thereby incrementing the magnon propagation length.

## Figures and Tables

**Table 1:** Key attributes and challenges for leading magnetic materials for magnonic applications. Information sources: YIG [39,40,41], Co_25_Fe_75_ [18,42], and MgAl_0.5_Fe_1.5_O_4_ [19].

	Thin Film/Substrate	(YIG)Y_3_Fe_5_O_12_/ Gd_3_Ga_5_O_12_ (GGG)	Co_25_Fe_75_/ Si/SiO_2_	MgAl_0.5_Fe_1.5_O_4_/ MgAl_2_O_4_
Parameters				
Gilbert damping constant		α ≈ 6.7 × 10^−5^	α ≈ 2.1 × 10^−3^	α ≈ 1.5 × 10^−3^
Magnon propagation length		Several cm	(21 ± 1) µm	(0.6–0.9) µm
Magnetostatic exchange length		~17.6 nm	~3.4 nm	~20.5 nm
Thin film fabrication		Liquid phase epitaxy on single crystal (111) Gd_3_Ga_5_O_12_ with PbO-B_2_O_3_ flux 927 °C.	Sputter deposited on Si/SiO_2_ substrates at ambient temperature. Polycrystalline films, no post-deposition processing required.	Pulsed laser deposition at 450 °C on MAO substrates. Epitaxial growth required.
Challenges for magnonic applications		Growth is not CMOS compatible. Expensive substrates.	Reduce extrinsic factors that increment the intrinsic damping parameter.	PLD fabrication is challenging to scale up. Alternative substrates. Low M_s_.
